# How to Reduce Mental Health Burden in Health Care Workers During COVID-19?–A Scoping Review of Guideline Recommendations

**DOI:** 10.3389/fpsyt.2021.770193

**Published:** 2022-01-20

**Authors:** Theresa Halms, Martina Strasser, Miriam Kunz, Alkomiet Hasan

**Affiliations:** ^1^Medical Faculty, Department of Psychiatry, Psychotherapy and Psychosomatics, Bezirkskrankenhaus Augsburg, University of Augsburg, Augsburg, Germany; ^2^Medical Faculty, Department of Medical Psychology and Sociology, University of Augsburg, Augsburg, Germany

**Keywords:** mental health, COVID-19, healthcare workers, recommendations, resilience

## Abstract

The COVID-19 pandemic has posed an unprecedented demand and a huge burden for healthcare workers (HCWs) worldwide, with alarming reports of heightened mental health problems. To counteract these mental health challenges, guidelines and recommendations for the support of HCWs during the COVID-19 pandemic have been published. With this scoping review and guideline evaluation, we aim to provide a critical overview of these guidelines and recommendations and to guide policy makers in establishing respective surveillance and care programs. In summary, 41 articles were included in this review which were published between April 2020 and May 2021. Across all articles, the guidelines and recommendations could be clustered into four main categories: “Social/structural support,” “Work environment,” “Communication/Information,” “Mental health support.” Although there was substantial agreement across articles about the recommendations given, empirical evidence on the effectiveness of these recommendations is still lacking. Moreover, most recommendations were developed without involving different members of the target group (HCWs) or other involved stakeholders. Strategies to detect potential barriers and to implement these guidelines in clinical practice are lacking.

## Introduction

The COVID-19 pandemic has posed an unprecedented demand and a huge challenge for healthcare workers (HCW), including physicians, nurses, interns, allied health professionals and other people working in the healthcare sector, worldwide for more than a year now. A meta-analysis (including 117 studies) investigating the impact of viral pandemics or epidemic outbreaks on HCWs' mental health showed increased levels of anxiety, depression and PTSD in HCWs during and after the outbreaks (1), which were associated with younger age, female gender, lack of social support, working in a high-risk environment and limited job experience (amongst others) (1). Similar findings were reported in another review article focusing exclusively on COVID-19, which showed that poor mental health outcomes were higher in nurses and were linked to inadequate personal protective equipment (PPE), fear of infection and heavy workload (2). Given these alarming reports, the question has been voiced of what can be done to protect HCWs and to reduce the risk of mental health burden during pandemic outbreaks in this crucial target group.

So far, numerous researchers, scientific institutions and health facilities have come forward with recommendations and guidelines on how to provide mental health support for HCWs and to mitigate the negative psychological outcomes of the COVID-19 pandemic. These recommendations range from minor suggestions to complex interventions and differ greatly in the underlying evidence. Due to the lack of scientific studies investigating the effectiveness of the suggested interventions and recommendations, it is uncertain whether they are indeed beneficial to HCWs. Little is known to which extent these guidelines are evidence or consensus-based or even representative. Non-evidence-based guidelines without formal consensus-processes have a significant risk of bias regarding the development of selected recommendations by specific stakeholder groups driven by individual conflicts of interest (3). Moreover, guidelines and recommendation papers can be considered instruments of quality management of the healthcare system aiming at improving quality and effectiveness of diagnostic and treatment procedures (4). Based on this framework, we conducted this scoping review to provide a comprehensive overview on published guidelines and recommendations for the support of HCWs during the COVID-19 pandemic and to critically evaluate these. The overall goal is to provide a comprehensive overview of the available evidence in order to guide policy makers in developing surveillance and care programs to improve mental health in healthcare workers during the pandemic.

## Methods

The search for recommendations and guidelines for the support of HCWs during the COVID-19 pandemic was carried out performing a systematic search using the literature databases PubMed, Cochrane Library and EMBASE using the following keywords: “COVID-19,” “mental health,” “resilience,” “health personnel” and “recommendations.” The search was carried out in May 2021 and all articles included were published between April 2020 and May 2021. Articles were excluded if they did not focus on the support of HCWs during the COVID-19 pandemic, included secondary literature such as pre-existing guidelines and recommendations, were in a language other than English or German or did not include any recommendations or guidance. The present review has been registered with the Open Science Framework (OSF): https://doi.org/10.17605/OSF.IO/6E4XZ.

### Quality of the Guidelines

Two assessors independently evaluated the included articles using the AGREE II instrument. As stated in the instructions of the AGREE II instrument (5), specific items may not be applicable to particular guidelines. We had to adjust this instrument (which focuses on clinical guidelines) to the given context and thus excluded several items. Items 11, 16, and 21 were excluded due to their inapplicability to non-clinical guidelines and recommendation papers. Items 10, 13, 14, 17, 20, 22, and 23 were excluded as the in this assessment included articles do not meet the methodological or formal requirements needed in order to apply these items. Each item was rated on a 7-point scale (1- strongly disagree to 7- strongly agree). Scaled domain scores were calculated as percentages of the maximum possible scores, according to the AGREE II methodology, using the following formula: (obtained score-minimum possible score) / (maximum possible score–minimum possible score), where the “obtained score” is the sum of the appraisers scores for each domain [see paragraph IV. Scoring the AGREE II (5)]. Hence, the discrepancies between the two assessors were considered during the process of evaluation. As reported in other studies using the AGREE II instrument, we considered a value >60% using the modified scale as a sufficient quality score and a value >80% as a good quality score (6, 7).

### Content of the Guidelines

To provide an overview on the types of recommendations given, two assessors extracted the recommendations and grouped them into different categories and within each category, into different topics. During a mutual process, key topics were derived from the given recommendations and recommendations were assigned to their respective key topic. However, recommendations that were mentioned in <5 out of the included 41 articles and could not be assigned to any of the existing key topics were excluded from the presented overview. The type of categories and topics are reported, together with the total number of articles including each recommendation.

## Results

**Figure 1:**
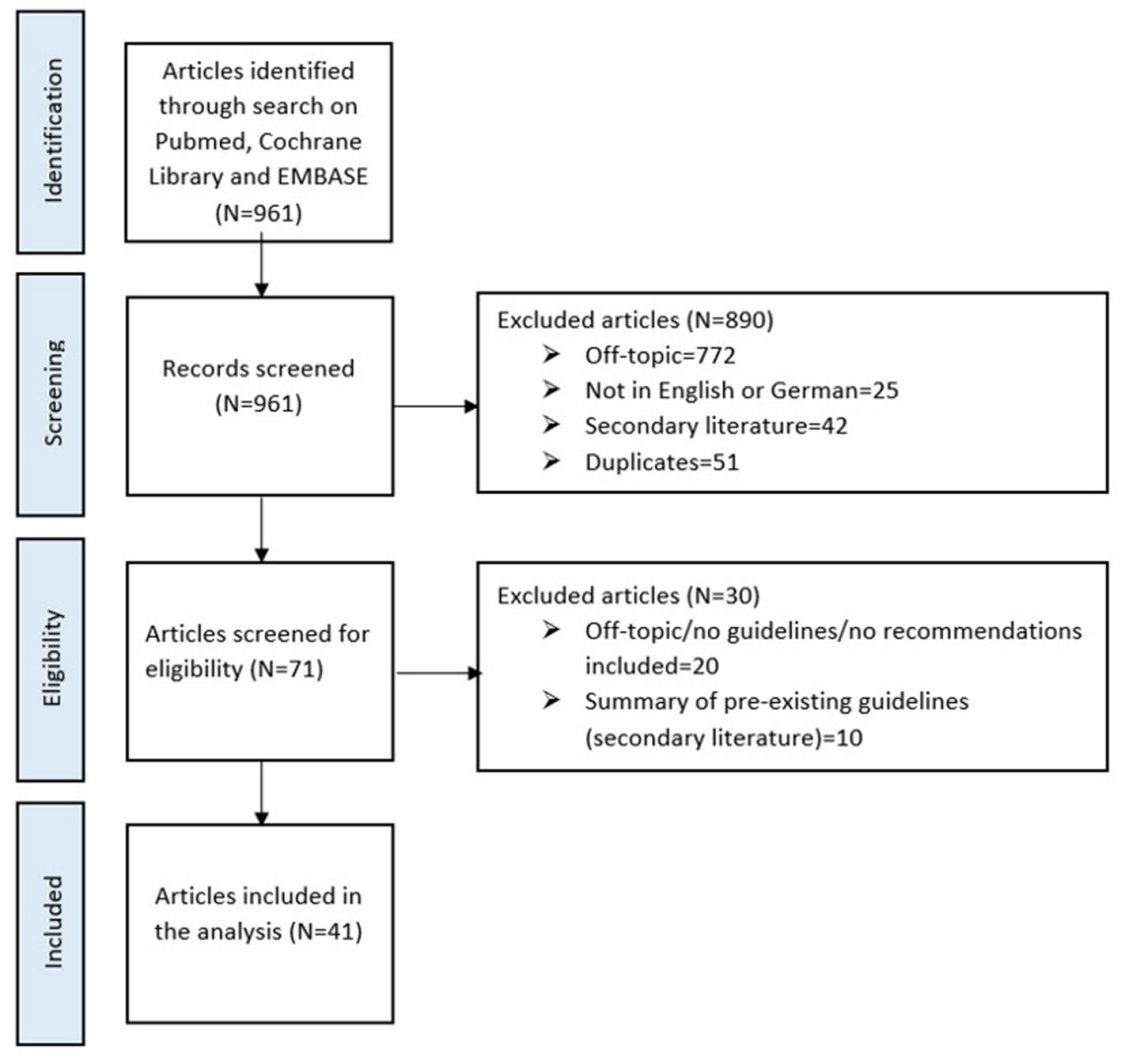
Flow chart of the literature search and selection of articles to be included in this review.

Figure 1 gives an overview of the article selection process. Most of the guidelines covered aspects and interventions on either an individual or an organizational level, whereas only a small number of articles focused on recommendations on a societal level.

### Quality of the Guidelines: Assessment *via* AGREE II

Table 1 provides a detailed overview of the outcomes per article.

**Table 1:** Domain scores calculated according to the AGREE II methodology for each of the included articles.

		**Scope and purpose**	**Stakeholder involvement**	**Rigor of development**	**Clarity and presentation**	**Application**	**Overall assessment**
**Authors**	**Title**						
Ahmed et al. (8)	How and when does inclusive leadership curb psychological distress during a crisis? evidence from the COVID-19 outbreak.	65.28%	66.67%	54.17%	54.17%	27.08%	54.17%
Albott et al. (9)	Battle buddies: rapid deployment of a psychological resilience intervention for health care workers during the COVID-19 pandemic.	91.67%	54.17%	54.17%	100.00%	79.17%	66.67%
Alnazly et al. (10)	Anxiety, depression, stress, fear and social support during COVID-19 pandemic among Jordanian healthcare workers.	83.33%	38.89%	14.58%	50.00%	16.67%	33.33%
Arnsten et al. (11)	Physician distress and burnout: the neurobiological perspective.	91.67%	36.11%	28.13%	58.33%	16.67%	41.67%
Awais et al. (12)	Paramedics in pandemics: protecting the mental wellness of those behind enemy lines.	69.44%	31.94%	17.71%	58.33%	35.42%	41.67%
Berkow et al. (13)	An executive strategy to support long-term clinician engagement amid the COVID-19 pandemic.	75.00%	47.22%	21.88%	62.50%	27.08%	45.83%
Billings et al. (14)	Supporting hospital staff during COVID-19: early interventions.	43.06%	34.72%	12.50%	70.83%	33.33%	33.33%
Boktor et al. (15)	Stress and anxiety management during the COVID-19 pandemic (lessons learnt from a cohort of orthopedic registrars redeployed to ITU).	72.22%	63.89%	20.83%	75.00%	20.83%	41.67%
Chew et al. (16)	Psychological and coping responses of health care workers toward emerging infectious disease outbreaks: a rapid review and practical implications for the COVID-19 pandemic.	94.44%	56.94%	67.71%	58.33%	37.50%	66.67%
Collins (17)	COVID-19: nurses have responded, now it is time to support them as we move forward.	65.28%	41.67%	15.63%	75.00%	45.83%	45.83%
Creese et al. (18)	“We all really need to just take a breath”: composite narratives of hospital doctors' well-being during the COVID-19 pandemic.	83.33%	55.56%	36.46%	25.00%	33.33%	45.83%
Donnelly et al. (19)	Well-being during coronavirus disease 2019: A PICU practical perspective.	76.39%	56.94%	28.13%	70.83%	56.25%	50.00%
Everly et al. (20)	Leadership principles to decrease psychological casualties in COVID-19 and other disasters of uncertainty.	77.78%	36.11%	27.08%	75.00%	41.67%	50.00%
Fukuti et al. (21)	How institutions can protect the mental health and psychosocial well-being of their healthcare workers in the current COVID-19 pandemic.	77.78%	50.00%	28.13%	75.00%	50.00%	50.00%
Gilleen et al. (22)	Impact of the COVID-19 pandemic on the mental health and well-being of UK healthcare workers.	77.78%	36.11%	18.75%	25.00%	8.33%	29.17%
Gray et al. (23)	A “mental health PPE” model of proactive mental health support for frontline health care workers during the COVID-19 pandemic.	94.44%	68.06%	32.29%	83.33%	70.83%	58.33%
Greenberg (24)	Mental health of health-care workers in the COVID-19 era.	69.44%	40.28%	25.00%	50.00%	37.50%	41.67%
Greenberg et al. (25)	How might the NHS protect the mental health of health-care workers after the COVID-19 crisis?	52.78%	27.78%	20.83%	25.00%	20.83%	33.33%
Hossain et al. (26)	Self-care strategies in response to nurses' moral injury during COVID-19 pandemic.	91.67%	41.67%	14.58%	83.33%	33.33%	41.67%
Hou et al. (27)	Social support and mental health among health care workers during coronavirus disease 2019 outbreak: a moderated mediation model.	76.39%	30.56%	53.13%	33.33%	29.17%	50.00%
Kamran et al. (28)	Effective recommendations for reducing anxiety and depression caused by COVID-19 outbreak in medical staff.	41.67%	25.00%	14.58%	70.83%	25.00%	33.33%
Karnatovskaia et al. (29)	Stress and fear: clinical implications for providers and patients (in the time of COVID-19 and beyond).	69.44%	51.39%	44.79%	91.67%	50.00%	58.33%
Kiser et al. (30)	When the dust settles: preventing a mental health crisis in COVID-19 clinicians.	47.22%	34.72%	10.42%	33.33%	12.50%	29.17%
Labrague et al. (31)	COVID-19 anxiety among front-line nurses: predictive role of organizational support, personal resilience and social support.	86.11%	69.44%	59.38%	50.00%	33.33%	66.67%
Li et al. (32)	Anxiety and related factors in frontline clinical nurses fighting COVID-19 in Wuhan.	91.67%	59.72%	32.29%	50.00%	37.50%	50.00%
Lissoni et al. (33)	Promoting resilience in the acute phase of the COVID-19 pandemic: psychological interventions for intensive care unit (ICU) clinicians and family members.	66.67%	52.78%	20.83%	58.33%	29.17%	45.83%
Markey et al. (34)	Cultivating ethical leadership in the recovery of COVID-19.	79.17%	51.39%	33.33%	41.67%	29.17%	45.83%
Miotto et al. (35)	Implementing an emotional support and mental health response plan for healthcare workers during the COVID-19 pandemic.	72.22%	47.22%	25.00%	50.00%	29.17%	45.83%
Orellano et al. (36)	Peruvian guideline to care the mental health of health providers during COVID-19 pandemic.	77.78%	25.00%	7.29%	58.33%	20.83%	29.17%
Owen et al. (37)	Leadership after a crisis: the application of psychological first aid.	61.11%	38.89%	16.67%	66.67%	29.17%	37.50%
Raudenská et al. (38)	Occupational burnout syndrome and post-traumatic stress among healthcare professionals during the novel coronavirus disease 2019 (COVID-19) pandemic.	83.33%	48.61%	40.63%	41.67%	16.67%	50.00%
Restauri et al. (39)	Burnout and posttraumatic stress disorder in the coronavirus disease 2019 (COVID-19) pandemic: intersection, impact, and interventions.	91.67%	50.00%	45.83%	91.67%	58.33%	62.50%
Ripp et al. (40)	Attending to the emotional well-being of the health care workforce in a New York City health system during the COVID-19 pandemic.	69.44%	61.11%	21.88%	75.00%	62.50%	58.33%
Schneider et al. (41)	Factors mediating the psychological well-being of healthcare workers responding to global pandemics: a systematic review.	75.00%	33.33%	32.29%	33.33%	50.00%	33.33%
Taylor et al. (42)	Mental health treatment for front-line clinicians during and after the coronavirus disease 2019 (COVID-19) pandemic: a Plea to the medical community.	51.39%	36.11%	25.00%	41.67%	54.17%	41.67%
Tomlin et al. (43)	Psychosocial support for healthcare workers during the COVID-19 pandemic.	88.89%	48.61%	32.29%	83.33%	54.17%	58.33%
Tracy et al. (44)	What should be done to support the mental health of healthcare staff treating COVID-19 patients?	73.61%	37.50%	20.83%	75.00%	45.83%	50.00%
Widjaja et al. (45)	Health issues among healthcare workers during COVID-19 pandemic: a psychosomatic approach.	63.89%	41.67%	36.46%	50.00%	33.33%	41.67%
Wilson et al. (46)	Caring for the carers: ensuring the provision of quality maternity care during a global pandemic.	86.11%	38.89%	18.75%	75.00%	45.83%	41.67%
Wong et al. (47)	Healing the healer: protecting emergency health care workers' mental health during COVID-19.	65.28%	51.39%	37.50%	83.33%	54.17%	50.00%
Wu et al. (48)	COVID-19: peer support and crisis communication strategies to promote institutional resilience.	66.67%	44.44%	25.00%	58.33%	33.33%	45.83%

#### Scope and Purpose

This domain evaluates whether the main objectives and the target population were specifically described. The median score of the scope and purpose domain was 75.00% (range 42–94%). Most articles described their overall objectives, questions and target populations sufficiently, however, five articles scored below the pre-specified value of 60%, which we considered to be the threshold value of a sufficient quality score.

#### Stakeholder Involvement

This domain assesses whether the guideline was developed by including individuals from all relevant professional groups. The median score of this domain was rather poor and reached only 44.44% (range 25–69%). Only five articles scored above 60% in this domain and very few articles considered the views and preferences of the target population (namely HCWs).

#### Rigor of Development

This domain assesses the quality of the evidence underlying the recommendations. The median score of the domain rigor of development was very poor and only reached 25.00% (range 7–67%). Apart from one article, all articles scored below 60%.

#### Clarity and Presentation

This domain evaluates whether the recommendations are specific and unambiguous. The median score of this domain was 58.33% (range 25–100%). Approximately half of the included articles scored under 60%.

#### Application

This domain focuses on factors related to guideline implementation. The median score of this domain was 33.33% (range 8–79%). Out of the included articles, 38 received scores under 60%. Most of the guidelines failed to describe facilitators and barriers to the implementation of the suggested recommendations.

#### Overall Assessment

This assessment requires a judgement as to the overall quality of the guidelines. Overall, the guidelines achieved a mean score of 46.24% (range 29–67%). Out of the included articles, 37 scored below the 60% mark. Hence, according to the assessment, only four guidelines would fulfill methodological standards to reduce the risk of bias.

### Content of the Guidelines: Types of Recommendations Given

**Figure 2:**
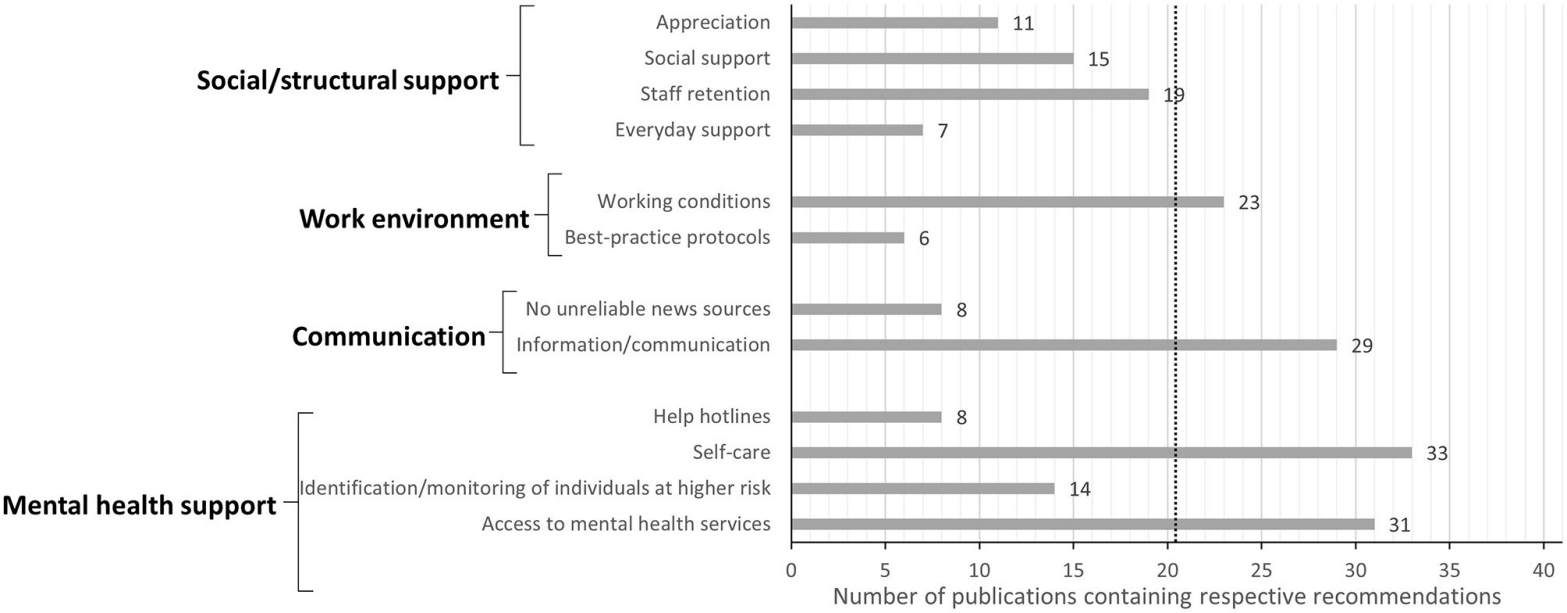
Overview of the types of recommendations given to improve mental health in HCWs during the COVID-19 pandemic. The numbers of publications including each recommendation are displayed. Dotted line displays the threshold of referencing the given topic in ≥ 50% of the included publications. A total of *N* = 41 publications were analyzed.

Specific recommendations have been identified and were clustered into four different categories. These categories and their respective key topics are displayed in Figure 2. A detailed overview on which key topic was included in which article can be found in Table 2. Out of the included articles, physicians were mentioned as the target occupational group in 11 articles, while nurses were mentioned in 12 articles. Management employees were mentioned in only one article, as well as specialist interns and patients or family members of patients. Allied health professionals, such as midwives or paramedics, were among the target occupational groups in 5 articles. Unfortunately, the majority of the articles (21 out of 41) did not further specify the term HCWs.

**Table 2:** Overview of the key topics included in each publication.

		**Appreciation**	**Social support**	**Staff retention**	**Everyday support**	**Working conditions**	**Best-practice protocols**	**No unreliable news sources**	**Information/** **communication**	**Help hotlines**	**Self-care**	**Identification/ monitoring of individuals at higher risk**	**Access to mental health services**
**Authors**	**Title**												
Ahmed et al. (8)	How and when does inclusive leadership curb psychological distress during a crisis? evidence from the COVID-19 outbreak.			✓		✓							✓
Albott et al. (9)	Battle buddies: rapid deployment of a psychological resilience intervention for health care workers during the COVID-19 pandemic.		✓			✓	✓	✓	✓	✓	✓		✓
Alnazly et al. (10)	Anxiety, depression, stress, fear and social support during COVID-19 pandemic among Jordanian healthcare workers.				✓	✓					✓		✓
Arnsten et al. (11)	Physician distress and burnout: the neurobiological perspective.		✓	✓					✓		✓		
Awais et al. (12)	Paramedics in pandemics: protecting the mental wellness of those behind enemy lines.	✓		✓		✓	✓		✓		✓		✓
Berkow et al. (13)	An executive strategy to support long-term clinician engagement amid the COVID-19 pandemic.			✓		✓	✓		✓				
Billings et al. (14)	Supporting hospital staff during COVID-19: early interventions.		✓	✓		✓			✓		✓	✓	✓
Boktor et al. (15)	Stress and anxiety management during the COVID-19 pandemic (lessons learnt from a cohort of orthopedic registrars redeployed to ITU).			✓							✓	✓	✓
Chew et al. (16)	Psychological and coping responses of health care workers toward emerging infectious disease outbreaks: a rapid review and practical implications for the COVID-19 pandemic.	✓							✓		✓	✓	✓
Collins (17)	COVID-19: nurses have responded, now it is time to support them as we move forward.												
Creese et al. (18)	“We all really need to just take a breath”: composite narratives of hospital doctors' well-being during the COVID-19 pandemic.		✓	✓	✓	✓			✓		✓		✓
Donnelly et al. (19)	Well-being during coronavirus disease 2019: A PICU practical perspective.	✓							✓		✓		✓
Everly et al. (20)	Leadership principles to decrease psychological casualties in COVID-19 and other disasters of uncertainty.								✓				✓
Fukuti et al. (21)	How institutions can protect the mental health and psychosocial well-being of their healthcare workers in the current COVID-19 pandemic.		✓		✓	✓	✓		✓	✓	✓	✓	✓
Gilleen et al. (22)	Impact of the COVID-19 pandemic on the mental health and well-being of UK healthcare workers.					✓			✓		✓	✓	✓
Gray et al. (23)	A “Mental Health PPE” model of proactive mental health support for frontline health care workers during the COVID-19 pandemic.									✓	✓	✓	✓
Greenberg (24)	Mental health of health-care workers in the COVID-19 era.	✓							✓			✓	✓
Greenberg et al. (25)	How might the NHS protect the mental health of health-care workers after the COVID-19 crisis?	✓							✓			✓	
Hossain et al. (26)	Self-care strategies in response to nurses' moral injury during COVID-19 pandemic.	✓				✓			✓		✓		✓
Hou et al. (27)	Social support and mental health among health care workers during Coronavirus Disease 2019 outbreak: a moderated mediation model.		✓										
Kamran et al. (28)	Effective recommendations for reducing anxiety and depression caused by COVID-19 outbreak in medical staff.					✓		✓	✓		✓		
Karnatovskaia et al. (29)	Stress and fear: clinical implications for providers and patients (in the time of COVID-19 and beyond).										✓		
Kiser et al. (30)	When the dust settles: preventing a mental health crisis in COVID-19 clinicians.	✓	✓	✓									✓
Labrague et al. (31)	COVID-19 anxiety among front-line nurses: predictive role of organizational support, personal resilience and social support.		✓	✓		✓			✓		✓		✓
Li et al. (32)	Anxiety and related factors in frontline clinical nurses fighting COVID-19 in Wuhan.		✓	✓		✓		✓	✓		✓		
Lissoni et al. (33)	Promoting resilience in the acute phase of the COVID-19 pandemic: psychological interventions for intensive care unit (ICU) clinicians and family members.					✓			✓		✓		✓
Markey et al. (34)	Cultivating ethical leadership in the recovery of COVID-19.					✓			✓		✓		
Miotto et al. (35)	Implementing an emotional support and mental health response plan for healthcare workers during the COVID-19 pandemic.				✓	✓	✓		✓	✓	✓	✓	✓
Orellano et al. (36)	Peruvian guideline to care the mental health of health providers during COVID-19 pandemic.		✓	✓		✓		✓	✓	✓	✓	✓	✓
Owen et al. (37)	Leadership after a crisis: the application of psychological first aid.			✓					✓		✓		✓
Raudenská et al. (38)	Occupational burnout syndrome and post-traumatic stress among healthcare professionals during the novel coronavirus disease 2019 (COVID-19) pandemic.		✓	✓		✓					✓		✓
Restauri et al. (39)	Burnout and posttraumatic stress disorder in the coronavirus disease 2019 (COVID-19) pandemic: intersection, impact, and interventions.			✓		✓		✓	✓		✓		
Ripp et al. (40)	Attending to the emotional well-being of the health care workforce in a New York City health system during the COVID-19 pandemic.	✓	✓	✓	✓	✓			✓		✓		✓
Schneider et al. (41)	Factors mediating the psychological well-being of healthcare workers responding to global pandemics: a systematic review.		✓		✓	✓		✓	✓		✓		✓
Taylor et al. (42)	Mental health treatment for front-line clinicians during and after the coronavirus disease 2019 (COVID-19) pandemic: a plea to the medical community.		✓								✓	✓	✓
Tomlin et al. (43)	Psychosocial support for healthcare workers during the COVID-19 pandemic.	✓		✓				✓	✓	✓	✓	✓	✓
Tracy et al. (44)	What should be done to support the mental health of healthcare staff treating COVID-19 patients?								✓		✓	✓	✓
Widjaja et al. (45)	Health issues among healthcare workers during COVID-19 pandemic: a psychosomatic approach.			✓						✓	✓		✓
Wilson et al. (46)	Caring for the carers: ensuring the provision of quality maternity care during a global pandemic.			✓		✓		✓			✓		✓
Wong et al. (47)	Healing the healer: protecting emergency health care workers' mental health during COVID-19.	✓	✓	✓	✓	✓	✓		✓		✓	✓	✓
Wu et al. (48)	COVID-19: peer support and crisis communication strategies to promote institutional resilience.	✓							✓	✓	✓		✓

#### Category “Social/Structural Support”

Within this category, **four** key topics were identified. As displayed in Figure 2, several articles highlighted the importance of “appreciation” of HCWs by the employers and/or the general public and recommended to raise more awareness for this aspect. The second key topic features recommendations revolving around the “social support” of HCWs that should be given by a variety of sources, such as family, friends, partners or coworkers. Furthermore, 19 out of the included 41 articles recommended to implement “staff retention,” for example by ensuring adequate compensation, rotating staff, mixing skills or deprioritizing non-essential work projects. Recommendations aiming at everyday support of HCWs (e.g., by providing free transportation, offering more childcare and providing adequate accommodation) were included in seven articles.

#### Category “Work Environment”

Recommendations concerning the work environment of HCWs can be summarized into two key topics: “working conditions” and “best practice protocols.” Recommendations regarding “working conditions” were mentioned in more than half of the included articles (see Figure 2). Here, suggestions to create a safe and employee-oriented work environment were laid out and included aspects such as providing adequate personal protective equipment (PPE) as well as providing ethics education, leadership training to supervisors, specialized job training and promoting professional development. Other strategies frequently recommended to ensure a safe work environment were infection control training and avoiding non-specific and mandatory interventions. The second key topic focuses on best-practice protocols to ensure the safety of clinical procedures. Such protocols include mandatory measures for minimizing HCWs' risk of contracting and spreading the coronavirus.

#### Category “Communication”

The category “communication” again covers two key topics: “no unreliable news sources” and “information/communication.” The first topic refers to recommendations concerning the use of news sources and social media. The second topic refers to how crucial information should be best communicated between team members and supervisors to ensure reliable information transfer (this was mentioned very frequently in 29 articles, see Figure 2).

#### Category “Mental Health Support”

This category focuses on recommendations concerning the mental health support of HCWs during and after the pandemic. Recommendations within this category were summarized into four key topics (see Figure 2). One key topic was “help hotlines” intended to provide mental health support while maintaining anonymity. Furthermore, the early identification and the active monitoring of individuals who show early signs of mental illnesses or who are at higher risk of developing mental problems was mentioned frequently. Recommendations focusing on the access to mental health services (psychiatric care or occupational therapy) were also mentioned very often (see Figure 2). Recommendations on “self-care” were mentioned most frequently (33 of the included 41 articles). Here, strategies such as self-help groups, peer support and team cohesion as well as encouraging well-being practices on an organizational level were mentioned in more than 50% of the 33 articles. Other strategies to promote self-care included guidance on resilience, stress management and mental health, providing the opportunity to talk to staff members, practicing self-care on an individual level and psychoeducation as well as resilience-building training.

## Discussion

Given the potentially wide-ranging mental health impact of COVID-19, protecting HCWs from adverse psychological effects and promoting their mental health and general well-being is critical. Over the course of the last year, several articles have been published, which provide suggestions and guidelines on how to achieve this. To evaluate the quality of these recommendations and guidelines, we used specific domains of the AGREE II instrument. Given the relative novelty of the COVID-19 pandemic, it is not surprising that the given recommendations and guidelines only achieved lower scores in the domains “rigor of development” and “application,” while moderate to high scores were achieved in the domain “scope and purpose.” The low scores can surely be explained by the dynamics of the pandemic that have not allowed for empirical investigations assessing the usefulness of the various recommendations. Overall, very few of the included articles laid the sole focus on the provision of recommendations, but rather presented them as a segment of their work. However, it has been recognized that preserving and improving mental health, resilience and well-being of HCWs poses a challenge that is influenced by environmental, structural, individual and team characteristics. (6). Therefore, we present a short but systematic overview of published recommendations on how to possibly strengthen mental health among HCWs during the COVID-19 pandemic.

### Mental Health Support

In most of the selected publications, authors emphasized the need for promoting better **self-care** of HCWs during this pandemic. Indeed, a basic component is meeting physical daily needs, such as supplementation of healthy nutrition and hydration, fitness, rest, and sleep. In the current setting (e.g., shortage of staff and time), these self-care aspects might often fall short for HCWs. Amongst the strategies for self-care practices on the individual level, diaphragmatic breathing (26, 28, 29, 32, 43), meditation (42, 43), maintaining a positive mind set (16, 29), mindfulness-, relaxation-, and problem-solving training (16, 26, 29, 32, 36, 39, 43, 45, 47) as well as maintaining personal interests, activities, and the connection to loved ones (16, 28, 32, 36, 43, 45–47) were mentioned frequently. Interestingly, avoiding maladaptive coping strategies (e.g., excessive alcohol consumption, overeating and prescription drugs) were only mentioned in two articles (46, 47). Interventions to encourage self-care on the organizational level included well-being courses, yoga or gym classes (12, 22, 23, 40) and providing opportunities for staff to talk about their experiences to enhance support and team cohesion (9, 14, 19, 43, 46). As reviewed, these recommendations on self-care are quite diverse and affect physical, psychological and social well-being of HCWs. It is possible that employees may not take up or use these offers due to lack of time or motivation after a long work shift. Strategies to overcome these potential barriers were not discussed in most articles. Finally, one must note that mental conditions like anxiety or depression itself of a certain degree can also result in barriers for the affected individual to promote self-care.

Recommendations addressing access to **mental health services** consist of (1) early identification of “at-risk” individuals (due to pre-existing experiences or mental health issues) so that plans can be put in place to support them, (2) actively monitoring anyone who has been exposed to a potentially traumatic event, (3) available access for staff members to psychologic or psychiatric support (e.g., helplines, online self-help programs, trauma focused PTSD treatment) (8–10, 12, 14–16, 19–24, 26, 31, 33, 35–38, 40–48). Providing psychological care and monitoring staff who are at higher risk of developing a mental disorder **after** the pandemic begins to recede were recommended in only five articles (14, 18, 24, 25, 37). Most guidelines seem to neglect the potentially ongoing stress and burden HCWs might face after pandemic (e.g., postponed surgeries and treatments, structural changes in the healthcare systems, staff shortage). Barriers and limiting factors for the use of mental health services, such as lack of anonymity or accessibility, were scarcely addressed in the included articles.

### Social/Structural Support

Interventions to improve mental well-being through social and structural support were also mentioned across many articles, which overlap to some extent with the strategies and topics mentioned above. Authors stressed the pivotal role of an appropriate **appreciation**, acknowledgment**, and professional validation** within the team and in particular as an integral part of the leadership style (12, 16, 19, 24–26, 30, 40, 43, 47, 48). Individual strategies include basic rules for respectful interaction, such as “giving thanks” (29). However, the majority of authors remained vague about specific strategies to actively show and promote appreciation, acknowledgment and validation. Organizationally, leaders are required to listen, learn and act (34). Not only in times of crisis is *an ethical, inclusive and effective leadership* required (e.g., leading by example, providing personal and professional support, involving staff in decision-making and action plans, establishing a human connection by validating an individual's feelings and thoughts) (8, 20, 34). Other strategies, such as providing free food and drinks, to show appreciation might not be sufficient. Authors highlight the role of **support**, both at the individual (e.g., family, friends, communities) and at the organizational level (peer support programs, online support, support from leaders). In respect to the recommendations concerning social support, it is noted that this aspect greatly depends on the support system and the resources of each individual. On that basis, the University of Minnesota Medical Center proposed an approach taken from the military framework. They developed a psychological resilience intervention founded on a peer support model (Battle Buddies) with 2 key elements: A Battle Buddy to provide peer support and a mental health consultant assigned to the unit (9).

### Communication

In times of crisis, it is important to provide **high-quality and transparent communication and accurate information updates** to all staff (14). Existing research shows that uncertainty leads to stress and anxiety (49). Stress increases with high work demands but co-occuring low work control (43). Therefore, leaders should provide staff with transparent and current updates so they are best prepared for what they are going to face and reflect on the risks and challenges (14, 43). That is especially important at the beginning of a crisis. Surprisingly, avoiding unreliable news sources and social media is recommended only by eight articles (9, 28, 32, 36, 39, 41, 43, 46). That raises the question whether the influence of social media and news is underestimated in this context. Authors further emphasize the need of **listening to staff input and feedback** (9, 11, 13, 14, 16, 19, 20, 28, 31, 33, 34, 36, 37, 40, 43, 44). Leaders should provide the opportunity to talk to them and implement regular feedback mechanisms. For implementation, it requires practical strategies. Once again, the shortage of time, exhaustion and staff shortage might be limiting factors.

### Work Environment

Authors often mentioned the need of adequate organizational support through the implementation of a safe and employee-oriented work environment. This includes the provision of **complete and quality Personal Protective Equipment (PPE) and supplies** to prevent infection, provision of accurate and timely information regarding the disease, employing best-practice protocols and guidelines, and implementation of **infection control trainings** (10, 12–14, 18, 21, 22, 31, 36, 38–41, 46, 47). Moreover, a few authors took into consideration that an employee-oriented work environment should promote professional development and provide specialized job training (16, 18, 22, 26, 35). Where possible, work environment should be optimized to support appropriate nutrition, rest (e.g., “take a minute” room) and sleep periods. While mandatory training and supervision programs (on the clinical skills required to deal with COVID-19 as well as on the potentially traumatic situations) might be beneficial for the team, some team members may have negative feelings and doubts toward mandatory interventions. Individual attitudes, preferences and sentiments might therefore have adverse effects on the team and counteract these interventions. This is not addressed in most articles. Another overlooked, yet crucial factor, is ensuring an adequate income as well as appropriate working hours for all occupational groups working in the healthcare system. While recommendations concerning these aspects might appear too obvious to be mentioned, it is necessary to stress their importance and potential consequences.

### General Remarks

Overall, recommendations on how to improve mental health in HCWs during the COVID-19 pandemic were targeted at various levels: from societal aspects to senior management and healthcare professionals. The relevance of protecting and promoting HCWs mental well-being must be viewed as a worldwide problem, as studies show the negative effects pandemics and epidemics have on the mental health of HCWs in several countries across Asia, Northern America, Middle East, Europe and West Africa (1). Additionally, a recent review showed an increase in the turnover intention in nurses in post-pandemic studies, posing the risk of further aggravating staff shortage (50). These circumstances can lead to a vicious cycle, putting more pressure on those remaining in their professions. Only few of the reviewed publications included intervention programs specifically designed to enhance mental health care for HCWs to face psychological challenges during the pandemic. Interestingly, most articles only focus on the time of the acute crisis and neglect what might happen when the crisis is over. However, we must be aware that the COVID-19 pandemic is rather a marathon, not a sprint (48). Against this background, we were surprised not to find a relevant number of recommendations regarding the prevention of mental health burden after the end of the pandemic. In that regard, only few of the reviewed publication described strategies and interventions to support HCWs' mental health after the crisis. Another pivotal, but so far neglected factor might be an adequate income for HCWs. Without ensuring a fair income, there might be little incentive to choose the profession and this in turn might cause staff shortage in the future. The potential consequences include e.g., higher workloads and more working hours for HCWs in the future, which might affect the work-life balance and cause even more physical and mental strain. Breaking this vicious circle is long overdue and should be addressed by political decision-makers. Government should provide healthcare organizations with sufficient resources (and appropriate income) to implement recommendations that fit their needs and adapt them to their context.

This review should be considered in light of some limitations. First, available publications for this review varied greatly regarding the publication type, which makes direct comparisons difficult and prevented us from conducting a formal systematic analysis. As reported by some of the authors, few of the recommendations and suggestions have substantial empirical evidence to support them. Second, our initial search may have neglected certain terms, however, re-inspecting our search by adding other potentially relevant key terms provided no additional articles suitable for our assessment. Further, we initially limited the search to the database Pubmed. Adding the databases Cochrane Library and EMBASE during the process of revision yielded in no additional publications. Moreover, we did not pre-specify our reported outcome categories and did not register this work in PROSPERO, as scoping reviews are not accepted for registration by PROSPERO. However, we instead registered our review on OSF Registries. The reason for our inductive approach was that no previous work was available, and we aimed at providing the very first overview assessing the quality of the guidelines. Furthermore, checking all included articles for the aspect of telemedicine showed that, surprisingly, only five articles mentioned telemedicine. This potential limited availability during a pandemic that may be derived from the underreporting in the selected publications, can be challenging for HCWs who are quarantined or who live in areas with limited access to mental-healthcare services. Next, the quality of the analyzed publication was low compared to standard medical guidelines. Especially the low scores for stakeholder involvement and rigor of development are a relevant source of bias.

Nevertheless, the urgency to develop specific psychological support intervention protocols for HCWs is apparent, not only in times of crisis. We believe that there are lots of measures that organizations, individuals, and national societies can take to minimize the impact of COVID-19 on the mental health of HCWs. However, it is essential to implementation some of the described measures to allow for early-detection and early-intervention in HCWs facing tremendous stress and burden. At this stage, one must conclude that despite a huge amount of available publications, evidence- and consensus-based guidelines on how to detect, prevent and treat psychiatric conditions in HCWs are lacking. The reasons for this gap were described in the previous paragraphs. While possible treatment options for HCWs with mental conditions during the pandemic can be extrapolated from available high-quality guidelines on how to treat e.g., anxiety, depression, trauma or alcohol dependency, more research is needed regarding the earlier detection and prevention in the vulnerable group of HCWs. Finally, in a pandemic, the perspective of different healthcare systems and cultures must receive far more attention.

## Conclusion

Our scoping review could identify four main categories of guidelines and recommendations to improve mental burden in health-care workers during the pandemic; namely “Social/structural support,” “Work environment,” “Communication/Information,” “Mental health support.” Although there was substantial agreement across articles about the recommendations given, empirical evidence on the effectiveness of these recommendations is still lacking. Moreover, most recommendations were developed without including the various members of the target group (HCWs) or other involved stakeholders. Thus, future recommendations should include these multi-disciplinary perspectives and hopefully will be able to also build a more solid empirical evidence base.

## Author Contributions

All authors listed have made a substantial, direct, and intellectual contribution to the work and approved it for publication.

## Funding

This work was funded by a grant of the Bavarian Ministry for Health and Care Services to MK and AH (Project mental health burden in health care workers during the COVID-19 pandemic: developing preventive strategies and offers of assistance).

## Conflict of Interest

AH is editor of the WFSBP and German schizophrenia guidelines. He received paid speakership from Janssen, Lundbeck, Recordati, and Otsuka and was member of respective advisory boards. The remaining authors declare that the research was conducted in the absence of any commercial or financial relationships that could be construed as a potential conflict of interest.

## Publisher's Note

All claims expressed in this article are solely those of the authors and do not necessarily represent those of their affiliated organizations, or those of the publisher, the editors and the reviewers. Any product that may be evaluated in this article, or claim that may be made by its manufacturer, is not guaranteed or endorsed by the publisher.
